# Study on the clinical correlation between the expression of serum TNF-α and iNOS as well as cognitive impairment and disease burden in patients with schizophrenia

**DOI:** 10.12669/pjms.38.7.5326

**Published:** 2022

**Authors:** Shouning Peng, Changcai Liang, Shimin Deng, Yanbo Fu

**Affiliations:** 1 Shouning Peng, Department of Psychiatry, Hainan Province Anning Hospital, Haikou 570206, Hainan, China; 2 Changcai Liang, Hainan Strong-arm Medical Clinic, Haikou 571146, Hainan, China; 3 Shimin Deng, Department of Psychiatry, Hainan Province Anning Hospital, Haikou 570206, Hainan, China; 4 Yanbo Fu, Department of Psychiatry, Hainan Province Anning Hospital, Haikou 570206, Hainan, China

**Keywords:** Schizophrenia, Tumor necrosis factor alpha, Inducible nitric oxide synthase, Cognitive impairment, Disease burden, Correlation

## Abstract

**Objectives:**

To investigate the clinical correlation between the expression of serum tumor necrosis factor-α (TNF-α) and inducible nitric oxide synthase (iNOS) as well as cognitive impairment and disease burden in patients with first-episode schizophrenia.

**Methods:**

A total of 102 patients with first-episode schizophrenia admitted to the Department of Psychiatry, Hainan Province Anning Hospital from June 2018 to June 2020 were randomly selected as the observation group, and 102 healthy people who underwent physical examination in our hospital during the same period were selected as the control group. Enzyme-linked immunosorbent assay (ELISA) was used to detect the expression level of serum TNF-α in each group, and the colorimetric method was adopted for detecting the expression level of serum iNOS in each group; Moreover, cognitive function of patients was assessed by MATRICS Consensus Cognitive Battery (MCCB), disease burden was assessed by Family Burden of Disease Scale (FBS), and the correlation between the expression of serum TNF-α and iNOS as well as cognitive impairment and disease burden was analyzed by *Pearson* method.

**Results:**

The serum levels of TNF-α, iNOS, trail making test scores and FBS scores of the observation group were significantly higher than those of the control group (*p<*0.05), while the scores of symbolic coding, verbal memory, maze, visual memory, and semantic fluency were significantly lower than those of the control group (*p<*0.05); *Pearson* correlation analysis results showed that the serum levels of serum TNF-α and iNOS in the observation group were positively correlated with the trail making test and FBS scores respectively (*p<*0.05), and negatively correlated with the scores of symbolic coding, verbal memory, maze, visual memory, and semantic fluency (*p<*0.05).

**Conclusions:**

Patients with first-episode schizophrenia have remarkably increased expression levels of serum TNF-α and iNOS, which is associated with cognitive impairment and disease burden.

## INTRODUCTION

Schizophrenia, as a clinically common severe mental illness, has a pathogenesis that is not yet fully clear, and has a certain correlation with heredity, environment, immune mechanism, and dopamine nervous system disorders. Schizophrenia has recurring episodes, and cognitive impairment is one of its important symptoms, bringing great pain and burden to patients and their families.^1^-^3^

Currently, the condition of schizophrenia is principally evaluated by the clinical manifestations of patients with such a disease, and there are still some difficulties in early judgment of the disease. Therefore, it is of great significance to find effective specific biomarkers for the diagnosis of schizophrenia. Tumor necrosis factor α (TNF-α) is an important pro-inflammatory factor that may play an important role in the pathogenesis of schizophrenia by stimulating nerve regeneration and maintaining an inflammatory state.^4^-^6^

Inducible nitric oxide synthase (iNOS) is the main rate-limiting enzyme of inflammatory response, which has a close bearing on the loss of dopaminergic neurons.^7^,^8^ However, few studies have been reported on the correlation between serum TNF-α and iNOS as well as cognitive impairment and disease burden in patients with schizophrenia. In view of this, in this study, the levels of serum TNF-α and iNOS in patients with first-episode schizophrenia were detected, and the correlation between them as well as cognitive impairment and disease burden was preliminatively investigated, so as to provide reference for the early diagnosis and treatment of schizophrenia.

## METHODS

A total of 102 patients with first-episode schizophrenia admitted to the Department of Psychiatry, Hainan Province Anning Hospital from June 2018 to June 2020 were randomly selected as the observation, including 54 males and 48 females, with an average age of (42.20±3.52) years, an average body mass index (BMI) of (21.56±2.29) kg/m2 and an average schooling years of (12.79±2.07) years. A total of 102 healthy people who underwent physical examinations in our hospital during the same period were selected as the control group, including 58 males and 44 females, with an average age of (41.93±3.11) years, an average BMI of (22.14±2.47) kg/m2 and an average schooling years of (13.11±1.65) years. The general information of the two groups of subjects was compared, see 2.1 Results for details.

### Ethical Approval

The study was approved by the Institutional Ethics Committee of Hainan Province Anning Hospital on February 22, 2021(202105), and written informed consent was obtained from all participants.

### Inclusion criteria:

- Patients meeting the diagnostic criteria for schizophrenia^9^;
- Patients with a total score of ≥60 points on the Positive and Negative Syndrome Scale (PANSS);
- Patients who had first onset and had not used any psychotropic drugs within 1 month before admission;
- Patients aged 18-55 years;
- Patients with junior high school education or above and good compliance;
- Patients who themselves and their family members knew and agreed to this study and signed the consent form.

### Exclusion criteria:

- Patients with organic brain diseases such as epilepsy and encephalitis;
- Patients with a family history of severe physical diseases and mental disorders;
- Patients with past craniocerebral trauma and diseases of heart, liver, kidney, endocrine, digestive system and autoimmune;
- Patients who abuse drugs or psychoactive substances;
- Patients with severe violent or suicidal tendencies;
- Pregnant or lactating women.

Detection of serum TNF-α and iNOS levels: 5 mL of fasting venous blood was taken from all subjects in the morning and centrifuged at 3000 r/minutes for 30 minutes to separate the serum. The level of serum TNF-α was determined by enzyme- Linked immunosorbent assay (ELISA), and the operation steps were strictly in accordance with the instructions of TNF-α ELISA kit (Shanghai Jianglai Biotechnology Co., LTD., Article No.: JL10208-96T). The level of serum iNOS was detected by the colorimetric method, and the operation steps were carried out strictly in accordance with the instructions of the iNOS kit (Nanjing Jiancheng Institute of Biological Engineering, Article No.: A014-1-1).

### Cognitive Function Assessment

The cognitive function of all subjects was assessed by two trained attending psychiatrists using the MATRICS Consensus Cognitive Battery (MCCB), among which six factors: trail making test, symbolic coding, verbal memory, maze, visual memory and semantic fluency were selected for assessment.

### Burden of Disease Assessment

The burden of disease was assessed by Family Burden Scale of Diseases (FBS), which included six dimensions of family economic burden, family daily life, family leisure and entertainment activities, family relationship, physical health of family members and mental health of family members. The higher the score, the heavier the burden.

### Statistical Analysis

All the data in this study were statistically analyzed using SPSS 22.0. Enumeration data were represented by number of cases (n), and chi-square test was performed. Measurement data were expressed as mean ± standard deviation (*X̅*±*S*), and t test was performed. *Pearson* correlation method was used for correlation analysis, and *p<*0.05 indicates a statistically significant difference.

## RESULTS

No statistically significant differences can be observed in age, gender, BMI and years of schooling between the observation group and the control group (*p>*0.05), as shown in Table-I. The levels of serum TNF-α and iNOS in the observation group were significantly higher than those in the control group (*p<*0.05). Table-II.

**Table I:** Comparison of general information between the two groups [(*X̅*±*S*)/n].

Group	Number of cases (n)	Age (years old)	Gender (Male/Female)	BMI (kg/m^2^)	Years of schooling (years)
Observation group	102	42.20±3.52	54/48	21.56±2.29	12.79±2.07
Control group	102	41.93±3.11	58/44	22.14±2.47	13.11±1.65
t/χ2 value	--	0.581	0.317	1.739	1.221
P value	--	0.562	0.574	0.084	0.224

**Table II:** Comparison of serum TNF-α and iNOS levels between the two groups (*X̅*±*S*).

Group	Number of cases (n)	TNF-α (pg/mL)	iNOS (U/mL)
Observation group	102	30.57±7.21	11.37±3.21
Control group	102	21.12±5.48	7.01±2.56
t value	--	10.539	10.725
P value	--	<0.001	<0.001

The trail making test score of the observation group was significantly higher than that of the control group, while the scores of symbolic coding, verbal memory, maze, visual memory and semantic fluency were significantly lower than those of the control group (*p<*0.05). Table-III.The FBS score of the observation group was significantly higher than that of the control group (*p<*0.05). Table-IV.

**Table III:** Comparison of cognitive function scores between the two groups (*X̅*±*S*).

Group	Number of cases (n)	Trail making test (points)	Symbolic coding (points)	Verbal memory (points)	Maze (points)	Visual memory (points)	Semantic fluency (points)
Observation group	102	53.21±14.03	43.39±11.13	16.86±4.54	11.02±3.54	16.35±4.57	16.27±5.35
Control group	102	35.36±8.12	50.17±15.02	25.61±6.29	20.17±6.74	25.82±9.19	34.11±.5.86
t value	--	11.121	3.663	11.392	12.138	9.319	22.707
P value	--	<0.001	<0.001	<0.001	<0.001	<0.001	<0.001

**Table IV:** Comparison of FBS score between the two groups (*X̅*±*S*).

Group	Number of cases (n)	FBS (points)
Observation group	102	23.62±6.21
Control group	102	7.47±2.15
t value	--	24.820
P value	--	<0.001

Pearson correlation analysis showed that serum TNF-α and iNOS levels in the observation group were positively correlated with the trail making test and FBS scores (*p<*0.05), and negatively correlated with symbolic coding, verbal memory, maze, visual memory and semantic fluency score (*p<*0.05). Table-V.

**Table V:** Correlation analysis of serum TNF-α and iNOS levels with cognitive function and FBS score.

Variable	TNF-α		iNOS	
	
	r value	P value	r value	P value
Trail making test	0.654	<0.001	0.589	<0.001
Symbolic coding	-0.640	<0.001	-0.599	<0.001
Verbal memory	-0.657	<0.001	-0.626	<0.001
Maze	-0.645	<0.001	-0.629	<0.001
Visual memory	-0.645	<0.001	-0.612	<0.001
Semantic fluency	-0.676	<0.001	-0.610	<0.001
FBS	0.663	<0.001	0.604	<0.001

## DISCUSSION

Schizophrenia is a severe mental illness of unknown etiology, which is often prolonged and recurrent and with a high disability rate, involving numerous disorders in terms of feeling, thinking, emotion and behavior. Cognitive impairment has become a characteristic core symptom of schizophrenia, bringing great pain and burden to patients and their families.^10^,^11^ It is not yet possible to judge schizophrenia based on specific pathological and physiological indicators. It has been shown in some studies that patients with schizophrenia suffer from obvious immune dysfunction and nerve damage in the body, and abnormal cytokine levels are associated with the onset of schizophrenia. It is therefore of great significance to search for effective biomarker indicators.

Previous studies have suggested that inflammatory response may exert an important role in the pathogenesis of schizophrenia. In current studies of schizophrenia, emphasis has been placed on inflammatory cytokines. They, together with neurotransmitters and endocrine hormones, constitute signal molecules between the cells of the body, with a wide range of central regulatory effects, which is closely related to psychological responses and mental disorders. Imbalance of cytokines will cause an increased risk of schizophrenia.^12^,^13^ TNF-α, as a kind of important inflammatory factors, bragging about promoting T cells to produce a variety of inflammatory factor, participating in the regulation, humoral immunity, and involve in schizophrenia stimulating nerve regeneration and maintaining an inflammatory state, thus playing a critical role in the pathogenesis of schizophrenia, immunity, nerve damage and other aspects.^14^,^15^ Patients with schizophrenia are in a state of immune activation, and their psychopathology is related to cytokines that regulate immune responses. It was shown in a study by Li QX et al.^16^ that the serum TNF-α concentration of patients with first-episode schizophrenia was significantly higher than that of healthy controls. As indicated by the results in this study, the level of serum TNF-α in the observation group was significantly higher than that in the control group, indicating that serum TNF-α, as an important cellular inflammatory factor, may be related to neurotransmitter and neuroendocrine, and has a negative effect on the nervous system. It not only has a regulatory effect on the nervous system, but also affects the balance between neuropeptides and neurotransmitters, thereby participating in the occurrence and development of schizophrenia.

Abnormal changes can be observed in the central and peripheral nitric oxide synthase (NOS) activity of patients with schizophrenia, which may lead to abnormalities in central dopamine and glutamate nervous system functions. It is believed in most current studies that schizophrenia has a close bearing on abnormal or unbalanced functional states of brain systems such as dopamine and glutamate.^17^,^18^ NO, as an important cellular active substance discovered in recent years, can cause a large amount of sodium and chloride ions to transfer into cells when it is produced in large quantities, resulting in nerve cell edema, cell membrane damage and neuron apoptosis in ischemic area. NOS is a key enzyme for the synthesis of NO. iNOS, by contrast, is the main rate-limiting enzyme and regulatory protein in the process of inflammation. It is closely related to the loss of dopaminergic neurons, and is mainly distributed in nerve cells, inflammatory cells, and glial cells. Moreover, iNOS is generally not expressed under normal physiological conditions, but continuous and substantial production of nitric oxide (NO) occurs in pathological conditions once induced synthesis.^6^,^7^,^19^ It was shown in this study that the level of serum iNOS in the observation group was significantly higher than that in the control group, suggesting that patients with schizophrenia may have abnormal expression levels of iNOS, which play an important role in the process of inflammatory response and abnormal nervous system function, and further participates in the occurrence and development of schizophrenia.

MCCB is a designated test for clinical trials of neuropsychiatric drugs, which can be used for cognitive assessment of schizophrenia and other neuropsychiatric disorders. Relevant studies have shown that patients with schizophrenia have a certain degree of cognitive dysfunction or impairment.^20^,^21^ In this study, the scores of trail making test in the observation group were significantly higher than those of the control group, while the scores of symbolic coding, verbal memory, maze, visual memory, and semantic fluency were significantly lower than those of the control group, indicating that the patients had a certain degree of cognitive dysfunction in the early stage of onset. *Pearson* correlation analysis showed that the levels of serum TNF-α and iNOS in the observation group were positively correlated with the trail making test scores, and negatively correlated with the scores of symbolic coding, verbal memory, maze, visual memory, and semantic fluency. It is speculated that the two may affect the cognitive function of patients with schizophrenia, and have a certain correlation with the cognitive impairment of patients.

Patients with schizophrenia, whose life care and guardianship responsibilities are mainly borne by their families, are often accompanied by a series of obvious thinking disorders and behaviors, such as labor capacity loss, long-term drug use, lengthy treatment cycle. However, the lack of a correct understanding of such a condition by family caregivers often leads to recurrence of the patient’s condition, a high recurrence rate, and even life-long treatment, which imposes a great long-term burden to the family on the material and spiritual aspect of the primary caregivers. FBS is a scoring standard commonly adopted at home and abroad for investigating the burden of patients with schizophrenia.^22^,^23^ As indicated by the results of this study, the FBS score of the observation group was significantly higher than that of the control group, indicating that schizophrenia may increase the burden of family diseases to a certain extent, and have a great impact on the family in terms of material, mental, and economic aspects. Further *Pearson* correlation analysis showed that the levels of serum TNF-α and iNOS in the observation group were positively correlated with FBS score, suggesting that the increase in the levels of serum TNF-α and iNOS may be associated with disease burden.

### Limitations of the study

Nevertheless, the mechanism of SERUM TNF-α and iNOS involved in cognitive impairment in schizophrenia is not completely clear and only preliminary studies have been carried out. For this reason, further in-depth studies are needed in the future to clarify the correlation between serum TNF-α, iNOS as well as cognitive impairment and disease burden, so as to provide a reference for the early diagnosis and treatment of schizophrenia.

## CONCLUSIONS

Patients with schizophrenia have up-regulated expressions of serum TNF-α and iNOS, which is associated with the cognitive impairment and disease burden of such patients. Moreover, the expression levels of serum TNF-α and iNOS may be used as potential biomarkers for evaluating the condition of schizophrenia.

### Authors’ Contributions:

**SP** & **CL:** Designed this study, prepared this manuscript, and are responsible and accountable for the accuracy and integrity of the work.

**SD:** Collected and analyzed clinical data.

**YF:** Data analysis, significantly revised this manuscript.
